# Searching for Errors in Models of Complex Dynamic Systems

**DOI:** 10.3389/fphys.2020.612590

**Published:** 2021-01-11

**Authors:** Dominik Kahl, Maik Kschischo

**Affiliations:** Mathematics and Technology, University of Applied Sciences Koblenz, Koblenz, Germany

**Keywords:** complex systems, open systems, fault detection, error localization, input reconstruction

## Abstract

Mathematical modeling is seen as a key step to understand, predict, and control the temporal dynamics of interacting systems in such diverse areas like physics, biology, medicine, and economics. However, for large and complex systems we usually have only partial knowledge about the network, the coupling functions, and the interactions with the environment governing the dynamic behavior. This incomplete knowledge induces structural model errors which can in turn be the cause of erroneous model predictions or misguided interpretations. Uncovering the location of such structural model errors in large networks can be a daunting task for a modeler. Here, we present a data driven method to search for structural model errors and to confine their position in large and complex dynamic networks. We introduce a coherence measure for pairs of network nodes, which indicates, how difficult it is to distinguish these nodes as sources of an error. By clustering network nodes into coherence groups and inferring the cluster inputs we can decide, which cluster is affected by an error. We demonstrate the utility of our method for the *C. elegans* neural network, for a signal transduction model for UV-B light induced morphogenesis and for synthetic examples.

## 1. Introduction

The dynamic systems we have to handle today, like ecological, biochemical or epidemiological networks, electric circuits or economic relations, are growing larger and more complex than they have ever been before (Kunegis, 2013; Rossi and Ahmed, 2015). The COVID-19 pandemic has highlighted one of the key limitations in our understanding of large and interconnected networks: An initially small disturbance can spread rapidly, which makes it difficult or impossible to reconstruct the root cause of the original perturbation. Modeling such processes is difficult in practice because it is hardly possible to understand each single interaction within a complex network, to monitor all external inputs, and to isolate the system from unwanted perturbations. In nearly all cases we have to deal with the presence of unknown *structural model errors*. We call these model errors *structural*, for they can lie in the functional form or in the very network topological structure of a system and can not be fixed by adjusting the parameters, compare (Engelhardt et al., 2016, 2017; Kahl et al., 2019; Villaverde et al., 2019).

Structural model errors impair the prediction of the future evolution of the system and also the estimation of the state from measured outputs. If the model is not trustworthy, it becomes questionable, whether the mathematical model reflects the reality at all (Tsigkinopoulou et al., 2017). Like in the pandemic, where the backtracking of infection chains plays a central role to keep the virus spreading under control—or at least to keep in sight—the reconstruction of structural model errors would aid manifoldly: We could recover the system's state, the location of the structural model error, and understand, which part of the system is affected. If we could infer a quantitative or qualitative description of the model error, we might be able to gain knowledge about the origin of the error.

Closely related to the problem of structural model errors is the theory of fault detection, an important topic in the engineering literature, see for instance Isermann (2011) and Blanke et al. (2016) for textbooks on fault detection, Fonod et al. (2014) and Chakrabarty et al. (2017) for works on unknown input observers. Geometrical and algebraic (Sain and Massey, 1969; Hirschorn, 1979; Fliess, 1988) treatments of the theory behind unknown input observers and fault detection of linear and non-linear systems have found renewed interest (Martinelli, 2019; Villaverde et al., 2019). A fault of the system, e.g., a mutation impairing a chemical reaction in a signal transduction network, can be seen as an endogenous model error. The practically most common approach to fault detection is to utilize *unknown input observers*. But those make strong assumptions about the system and especially about the ability to precisely understand the interactions and to collect data, see for instance Fonod et al. (2014) and Chakrabarty et al. (2017). These assumptions may be justified for systems which went through a design process, but they become questionable as soon as we work with biological systems like a cell or even an organ, which permanently interact with their exterior, whereas we do not even oversee the vast number of internal processes.

Dynamic systems in continuous time are often formulated as systems of ordinary differential equations,

(1) $$\begin{array}{l} \;\overset{∙}{x}\left(t\right)=f\left(x\left(t\right),t\right)\\ x\left(0\right)=x_{0}\\ y\left(t\right)=c\left(x\left(t\right)\right), \end{array}$$

where ***x***(*t*) ∈ ℝ^*N*^ represents the current state of the system at time *t*, ***x***_0_ is the initial state, and **y**(*t*) ∈ ℝ^*P*^ represents the directly observable outputs at time *t*. Often we are not able to monitor each and every state variable *x*_*i*_, but we can only measure some state variables or combinations of them. As the observables are the only experimentally accessible quantities of the system, any knowledge about the system can only be inferred from a comparison between the model output **y** and the measured data **y**^data^. Henceforth, we assume that we have experimental data **y**^data^ for the system of interest, whereas **y** describes the output expected from our theoretical model (1). In reality, the system might be affected by structural model errors, i.e., a mis-specification of the vector field **f** encoding the interactions between the state variables. As a consequence, data and model output do not coincide, **y**^data^ ≠ **y**. Due to this discrepancy, conclusions and predictions based on the model (1) can be incorrect or inaccurate.

Algorithms for recovering the errors of a given model (1) from data implicitly assume that the root cause for an error can uniquely be identified (Kolodziej and Mook, 2005; Engelhardt et al., 2016, 2017; Villaverde et al., 2019; Newmiwaka et al., 2020). However, this uniqueness assumption is often violated because the output function **c** does not provide sufficient information (Engelhardt et al., 2016; Kahl et al., 2019) about the root cause for the observed discrepancy **y** − **y**^data^ between model and data. Based on the representation of model errors as unknown inputs (Kolodziej and Mook, 2005; Engelhardt et al., 2016) to the system (1), it was possible to relate the problem of error reconstruction to the problem of system invertibility (Kahl et al., 2019). This enabled us to utilize algebraic and graph theoretic results (Fliess, 1988; Wey, 1998) to derive conditions on the system (1), which guarantee the unique reconstruction of model errors (Kahl et al., 2019, 2020). The analysis of biological systems revealed that the unique reconstruction of model errors is often difficult, unless the measurements [i.e., **c** in (1)] are carefully selected (Kahl et al., 2019). This lead to a powerful sensor node placement algorithm, which can drastically improve the invertibility properties of a given system (Kahl et al., 2019). However, this algorithm is restricted to the case that we know already the potential *location* of the error in the system.

In this paper, we present a strategy to handle systems for which it is impossible to pinpoint the exact state variables affected by model error. To the best of our knowledge, this problem has not been discussed before. First, we provide a new measure of *input coherence* between two different state variables in the system (1). This coherence quantifies, how difficult it is to decide which of the two state variables is targeted by an error with unknown location. The coherence measure is based on the concept of weighted gammoids as a representation for dynamic input-output systems. We use the pairwise coherence between all states in the system to group system states into *input clusters*. Whereas we are not able to recover the model error, we will at least be able to *localize* it up to the level of input clusters. In duality, we also form output clusters of coherent outputs. This will help to identify redundant measurements and to select new observables that yield complementary information. Finally, we show that an iterative strategy of clustering error sources and sensors can narrow down the possible error sources successively until we, in the best case, are able to pin it down exactly.

## 2. Methods

### 2.1. Background on Input-Output Networks and the Reconstruction of Model Errors

If the system (1) does not correctly describe the observed data **y**^data^, it is affected by a structural model error. Such errors include endogenous errors like missing or wrongly specified interactions in the vector field **f** as well as exogeneous errors originating from interactions of the system with the environment. Mathematically, all these errors can be represented by unknown inputs ***w***(*t*) acting additively on the vector field (Engelhardt et al., 2016; Kahl et al., 2019). Thus, we modify (1) to

(2) $$\begin{array}{l} \overset{∙}{x}\left(t\right)=f\left(x\left(t\right)\right)+w\left(t\right)\\ x\left(0\right)=x_{0}\\ y\left(t\right)=c\left(x\left(t\right)\right). \end{array}$$

Can we reconstruct the error ***w***(*t*) from the measured output data **y**^data^? A unique reconstruction of the error requires that there is only one possible input function $w\left(t\right)={\left(w_{1}\left(t\right),\ldots ,w_{N}\left(t\right)\right)}^{T}$ generating the measured output **y**^data^. This means, that the input-output map Φ:***w*** ↦ **y** needs to be injective (one-to-one).

Assume for the moment that we know, which state variables are affected by an error. This means that we know at least the non-zero components of the underlying true model error w^*^(*t*) which we are aiming to reconstruct. If we denote the index set of all the states ***x*** ∈ ℝ^*N*^ in (2) as N={1,…,N} we will call the subset S⊆N of states affected by an error the *input set*. In a similar way, we define the *output set* Z⊆N of state variables appearing in the output function **c**(***x***). Mathematically, this means that *i* ∈ *Z* is equivalent to $\frac{\partial c_{j}\left(x\right)}{\partial x_{i}}\neq 0$ for some output index *j* ∈ {1, …, *P*} and at some state ***x***. The output nodes in *Z* are also often called *sensor nodes* in the literature and we will use these terms synonymously (Liu and Barabási, 2016).

It turns out that under the (unrealistic) assumption of zero measurement noise in the data, the question of unique error reconstruction can be answered from a purely graphical condition (Kahl et al., 2019). The vector field of a dynamic system of the form (1) or (2) can be represented by an *influence graph* *g* (see e.g., Lin, 1974; Dion et al., 2003; Boukhobza et al., 2007; Lunze, 2016). This directed graph *g* is formed by identifying the state variables *x*_*i*_ with the nodes N of *g*. The directed edge *x*_*i*_→*x*_*j*_ exists in *g* if *x*_*i*_ appears in the differential equation for *x*_*j*_, i.e., if ∂*f*_*j*_/∂*x*_*i*_ ≠ 0. The set of all such edges E in *g* indicates, which states interact with each other.

The condition for the unique recovery of the model error ***w*** for a known input set *S* is related to the invertibility (Sain and Massey, 1969; Fliess, 1988) of the dynamic system (2). One can derive a graphical condition for the invertibility (Wey, 1998) of a non-linear system and thus decide, whether the error ***w*** can uniquely be recovered (Kahl et al., 2019): If there is a set of node disjoint paths linking each node in the input set *S* with a node in the output set *Z*, then the system is invertible. From the graphical condition (Kahl et al., 2019) one can conclude a necessary condition on the minimum of number of sensor nodes, i.e., the cardinality *P* = |*Z*| of the output node set *Z*. For invertibility, we need |*Z* |≥| *S*|, i.e., at least as many measurements as inputs. However, this is not always sufficient. Many badly placed sensors can also prevent us from reconstructing the unknown input. A very efficient sensor node placement algorithm to select a minimum output set *Z* which guarantees invertibility is available (Kahl et al., 2019).

The graphical condition for model error recovery (Kahl et al., 2019) is limited to the case that the input set *S* is known and that there is no measurement noise. If the measurement data **y**^data^ are noisy, then we can ask whether we can minimize the error $∥y^{\text{data}}-\Phi \left(w\right){∥}_{2}^{2}$ between the output **y**(*t*) = Φ(***w***)(*t*) from (2) and the data with respect to the unknown input ***w***. However, one can show that even in the invertible case the inverse of the input-output map Φ is highly sensitive to measurement noise. Thus, the reconstruction requires a suitable regularization (Kahl et al., 2020).

The most challenging case is that the input set *S* is unknown and that the data **y**^data^ are corrupted by noise. Recently, we derived conditions that the minimization of the error functional

(3) $$\begin{array}{l} J\left[w\right]=\frac{1}{2}∥y^{\text{data}}-\Phi \left(w\right){∥}_{2}^{2}+\beta ∥w{∥}_{1}. \end{array}$$

recovers the correct unknown input w^*^ to a certain level of accuracy from the data (Kahl et al., 2020). These conditions, however, require two additional assumptions: The first one is *invariable sparsity* of the input ***w***. This essentially means that the unknown input set *S* is assumed to have a maximum cardinality and to be constant over time. This assumption, is, however, reasonable for structural model errors and also for system faults, because the location of an error or fault (not the error signal itself) is unlikely to change over time. This is to be distinguished from other definitions of sparsity in control theory, where the input location can jump between different states (Nagahara et al., 2016). The second, more severe, assumption for the recovery of the model error by minimizing (3) is the linearity of the input-output map Φ. This does not mean that the minimization of (3) or related cost functions (Kolodziej and Mook, 2005; Engelhardt et al., 2016) for non-linear systems is impossible. It only says that we have currently no guarantee to recover the correct model error w^*^(*t*).

In many cases, it is hard to decide whether the recovery of the true model error w^*^ is possible or not (Boukhobza et al., 2007; Villaverde et al., 2019; Kahl et al., 2020). As described above, it is sometimes useful to divide the problem into two parts: First, one needs to find the input set *S* and second one needs to recover the error signal ***w*** (Kahl et al., 2020). However, in this text we will take a slightly more pragmatic approach: For a given model (1) augmented by an unknown input (2), is it possible to narrow down the input set *S* to a smaller subset? That means, can we at least identify a region in the network (influence graph *g*) which is affected by the model error?

### 2.2. Gammoids and Coherence of Dynamic Systems

For a given system with errors or unknown inputs, we assume that we have at least the influence graph *g*, the output set *Z* and time course data **y**^data^(*t*) for these data. Typically, these are taken at discrete time points *t*_1_, …, *t*_*T*_. In addition, we might have weights *F*(*i* → *j*) indicating the strength of the interaction between the states ***x***_*i*_ and ***x***_*j*_. For a dynamic model (12) we can obtain the weights from the Jacobian

(4) $$\begin{array}{l} F\left(i\to j\right):=\frac{\partial f_{j}}{\partial x_{i}}\left(x^{\left(r\right)}\right) \end{array}$$

at a certain reference point ***x***^(*r*)^. This reference point could, for instance, be a stationary state or an initial condition. The weights might also be obtained from other sources, see our example in section 3.1.

If the input node set *S* is unknown, we have to consider all nodes N in *g* as potential input nodes. However, unless we measure all states or make further assumptions, we can not reconstruct the location *S* of the unknown input (Kahl et al., 2019). To isolate at least the regions in the network *g*, where errors or inputs are located, we need a measure of independence between potential input nodes L⊂N. We will refer to this set L of potential input locations as the *ground set*. If we have some prior information on the location of the error, the ground set could be a proper subset of the set of state nodes N. However, in the absence of any prior knowledge, L=N is also possible in our approach.

The available structural information can be collected in a mathematical structure called a *gammoid*.

** DEFINITION 1**. *Let* g=(N,E) *be the influence graph of a dynamic system (2),* L⊆N *a*n *input ground set* *and* Z⊆N *an output set. We call* Γ:=(L,g,Z) *the* *gammoid* *of the system*.

The gammoid of the system combines all possible input sets into one structure. The idea of the input ground set L is that it comprises all allowed input nodes, such that each subset S⊆L serves as a candidate for the true input set. If we combine the weight function F:E→ℝ given by (4) with the gammoid Γ, one can show that we obtain a so called weighted gammoid (Kahl et al., 2020). Thus, a weighted digraph with inputs and outputs can be regarded as a weighted gammoid.

The advantage of the additional abstraction to gammoids is the fact, that they give rise to an abstract independence structure on the input ground set (Perfect, 1968; Pym, 1969a,b; Mason, 1972). This independence structure can be used to derive conditions on the recovery of invariable sparse inputs to non-linear systems in the case that the input set *S* is unknown (Kahl et al., 2020). These conditions are, however, still restricted to perfect data **y**^data^ without measurement noise. It does not make any statements about the numerical stability of a numerical recovery algorithm like minimizing (3). Here we will exploit the independence structure to detect groups of potential input nodes in L which can not be distinguished from any measured output data **y**^data^ acquired at the sensor nodes *Z* of the gammoid.

#### 2.2.1. Concatenation of Gammoids

For a gammoid Γ=(L,g,Z) we can introduce the *transposed gammoid* $\Gamma^{\prime }:=\left(Z^{\prime },g^{\prime },L^{\prime }\right)$ in the following way (Kahl et al., 2020): For each node i∈N we introduce a node $i^{\prime }\in N^{\prime }$. Here, the prime helps to distinguish the nodes in g=(N,E) and the transposed graph $g^{\prime }=\left(N^{\prime },E^{\prime }\right)$. Then, *g*′ is obtained from *g* by flipping all the edges and the corresponding weights. Thus, for each edge (i→j)∈E there is a flipped edge ${\left(i\to j\right)}^{\prime }=\left(j^{\prime }\to i^{\prime }\right)\in E$. Accordingly, the weights of Γ and Γ′ are related by *F*(*i*→*j*) = *F*(*j*′ → *i*′). At the same time, also the ground set L and the output set change roles in $\Gamma^{\prime }:=\left(Z^{\prime },g^{\prime },L^{\prime }\right)$. The output nodes *Z* in Γ correspond to the inputs of Γ′ and the inputs of Γ to the outputs of Γ′.

Then, one can concatenate Γ with its transpose Γ′ by identifying each output node *z*_*i*_ ∈ *Z* with the corresponding input node $z_{i}^{\prime }\in Z^{\prime }$. Thereby we obtain again a gammoid, denoted $\Gamma \circ \Gamma^{\prime }=\left(L,g\circ g^{\prime },L^{\prime }\right)$, where *g* ∘ *g*′ is the resulting graph produced by identifying *Z* with *Z*′.

#### 2.2.2. The Shortest Path Coherence and Clustering of Similar Input Nodes

The composed gammoid Γ ∘ Γ′ will be used in the following to compute a measure of coherence between different potential inputs.

** DEFINITION 2**. *Let Γ be a weighted gammoid with ground set* $L=\left\{l_{1},\ldots ,l_{L}\right\}$. *For two nodes* $l_{i},l_{j}\in L$ *let* ψ_*ij*_ *denote the shortest path from* *l*_*i*_ *to* $l_{j}^{\prime }$ *in (Γ ∘ Γ′). We call*

(5) $$\begin{array}{l} \mu_{ij}^{short}:=\frac{\left|F\left(\psi_{ij}\right)\right|}{\sqrt{F\left(\psi_{ii}\right)F\left(\psi_{jj}\right)}} \end{array}$$

*the shortest path coherence between* *l*_*i*_ *and* *l*_*j*_.

The shortest path coherence can readily be computed even for large (*N* > 100) networks. One simply computes the shortest path between the two nodes and the corresponding weight and normalizes this according to (5).

The shortest path coherence can be used as a measure for the similarity of the output patterns that can be generated from state node *i* and *j*. The larger $\mu_{ij}^{\text{short}}$ is, the harder it is to decide from the output data **y**^data^, whether there is an input *w*_*i*_(*t*) at state *i* or an input *w*_*j*_(*t*) at *j*. An exact proof for this is beyond the scope of this paper, see our more theoretical exposition (Kahl et al., 2020). There, we also show how the shortest path coherence can also be used for testing, whether an error can exactly be localized, or not.

If there is a high pairwise shortest path coherence between different nodes, it is natural to combine these nodes into a group of highly coherent states. Thus, we try to identify clusters of nodes which are highly coherent to each other, i.e., where we can not decide, which of the states within the cluster is targeted by an error or unknown input. For clustering, it is often easier to work with a distance measure between the input nodes *i, j*. Thus, we define the *(shortest path) distance* as

(6) $$\begin{array}{l} d_{ij}:=\frac{1}{\mu_{ij}^{\text{short}}}-1\;\left(i,j\in \left\{1,\ldots ,N\right\}\right). \end{array}$$

The corresponding shortest path distance matrix *D* = (*d*)_*ij*_ can readily be used as a distance matrix for standard hierarchical clustering algorithms. We used complete linkage clustering, i.e., the distance between a node *i* and a cluster C is the maximum of *d*_*ij*_ for all j∈C. For our computations we used the python package networkx for graph theoretical algorithms and scipy and seaborn to perform a hierarchical clustering of the distance matrix. Sometimes it is helpful to rescale *D* and use the normalized variant $D/\max_{ij}\left(d_{ij}\right)$ for visualization purposes. It shall be noted that it is not unusual to find some shortest path coherences μ_*ij*_ to be zero or close to zero. This results in a divergence of the distance matrix. It has proven practical to work with an appropriate upper bound for the distances.

## 3. Examples

### 3.1. Coherences in the *Caenorhabditis elegans* Neural Network

**Figure 1:**
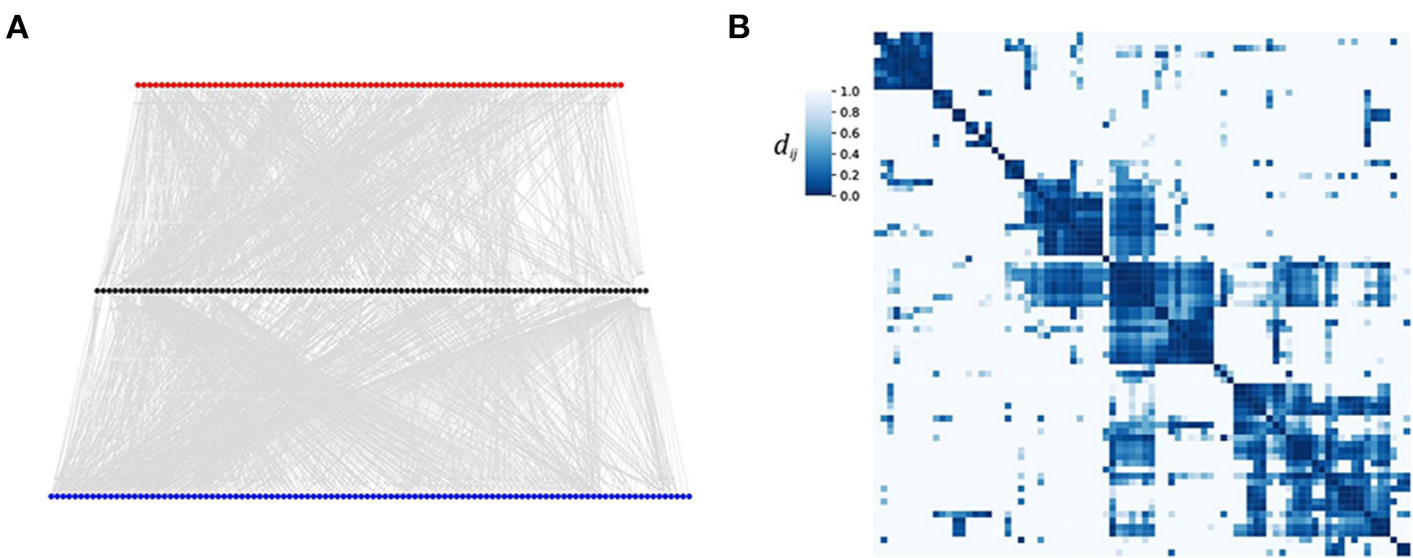
Neural input clusters for *C. elegans*. **(A)** The *C. elegans* connectome plotted in three layers of neurons [presented in Varshney et al., 2011, data set available (Altun et al., 2002–2020)]. Red nodes indicate sensor neurons, black nodes represent inter neurons, blue nodes are the motor neurons. **(B)** A heat map of the distance matrix *D* = (*d*_*ij*_) of the 82 sensor neurons. Nodes of low distance (high coherence) are difficult to distinguish by their outputs. Thus stimuli to nodes within the same cluster are likely to induce the same movements. We used a distance bound $\bar{d}=10$ and distance threshold $d_{\text{threshold}}=10^{-5}$.

*Caenorhabditis elegans* (*C. elegans*) is a small worm. Its connectome, i.e., the network of neurons and synapses was completely mapped and is available as a comprehensive resource (Altun et al., 2002–2020; Corsi et al., 2015) and a network scientific treatment of the *C. elegans* connectome in Varshney et al. (2011) and Yan et al. (2017). But, a neural structure like the one of *C. elegans* does not only offer a network interpretation. Due to the input-output structure it naturally induces a weighted gammoid: For the network interpretation, the neurons are the nodes and the synapses are directed edges which allow for a direct information transfer from one neuron to the following one. Some links comprise several synapses, so that we can take the number of synapses between two neurons *i* and *j* as the weight of (*i* → *j*). Finally, neurons are divided into sensor neurons which are sensible to inputs, inter neurons which process the input, and motor neurons which finally pass the processed information to the muscles and induce the locomotion of the worm. Please note, that sensor neurons are the inputs in this example. They should not be confused with the sensor nodes or outputs (here motor neurons). See Figure 1A for a view on the *C. elegans* neural network (Varshney et al., 2011) in a simplistic three layer illustration with the sensor neuron layer on top, the inter neuron layer in the middle, and the motor neuron layer at the bottom [data obtained from the worm atlas (Altun et al., 2002–2020)].

An individual of *C. elegans* is either of male sex or hermaphrodite. These two differ slightly in their connectome. Here, we used the hermaphrodite data with a total node number of *N* = 283. Figure 1B presents a heat map of the distance matrix obtained from the shortest path coherence of the 82 sensor neurons of *C. elegans* already sorted into clusters. We find that the input ground set, i.e., the sensor neurons, fall into few (four to five) large clusters which cover most of the 82 input nodes. Inputs to nodes within the same cluster cannot be distinguished by the outputs of the network. Thus, stimuli targeting sensor neurons of the same cluster are likely to generate the same signal in the motor neurons and thus the same movement. This result is consistent with earlier work (Stephens et al., 2008), where it was shown that 95% of the worm's locomotion can be described by only four characteristic movements.

This example illustrates the ability of our gammoid approach to define highly coherent input nodes, i.e., nodes which will induce the same outputs. In this way, the coherence clustering can be used to classify the set of potential output patterns and to investigate the generalizing effect characteristic for neural networks. In principle, the same approach can be used for other directed and weighted networks.

### 3.2. Cluster Localization of Perturbations in a Model for Signal Transduction in Response to UV-B Light

In this example we use an ODE based model of a signal transduction network for the induction of photomorphogenesis by UV-B light (Ouyang et al., 2014). There are 11 state variables *x*_1_, …, *x*_11_ and five outputs *y*_1_, …, *y*_5_, see the Supplementary Material for the equations. We first generate pseudo-experimental data for a system that is perturbed by two model errors. We randomly chose $w_{3}^{*}$ and $w_{6}^{*}$ acting on the state variables *x*_3_ and *x*_6_, respectively. The error signals $w_{3}^{*}\left(t\right)$ and $w_{6}^{*}\left(t\right)$ are documented in the Supplementary Material. We initialized the model close to the stationary point by setting the initial value to ***x***^(*r*)^ = (1.89, 0.17, 0.0007, 34.34, 1.63, 0.048, 0.098, 2.27, 0.40, 8.17, 11.82)^*T*^ and added Gaussian measurement noise to each measurement *y*_*i*_(*t*_*k*_) with a relative standard deviation of 5% of *y*_*i*_(*t*_*k*_). Here, *t*_*k*_ is the time of the *k*-th data point.

**Figure 2:**
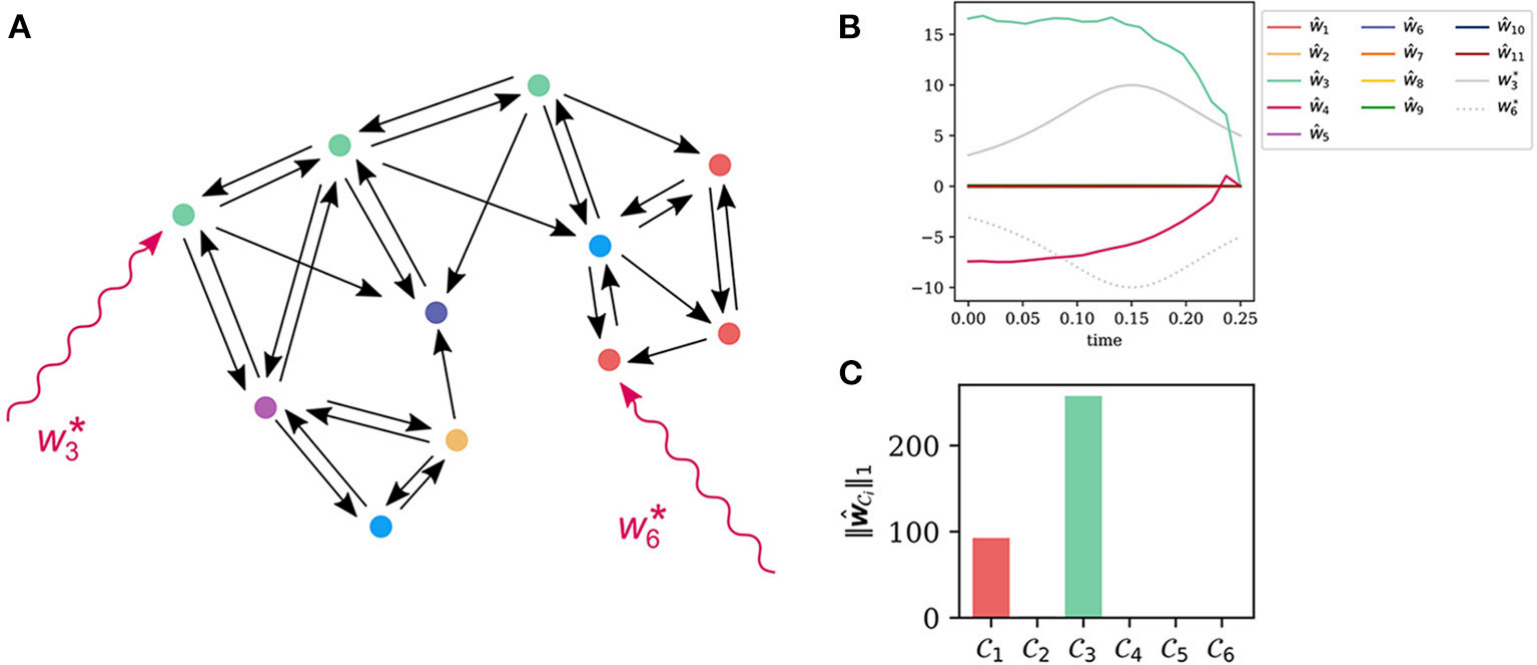
Cluster based localization of unknown inputs in a signal transduction model of UV-B light induced photomorphogenesis (Ouyang et al., 2014). **(A)** The influence graph for the model with 11 state variables. The states *x*_6_ and *x*_3_ are targeted by a simulated model error. Computing the coherence matrix (5) at a stationary point and clustering yields six input clusters $C_{1},\ldots ,C_{6}$. For state nodes within the same input cluster it is difficult to distinguish them as the sources of the error. **(B)** The time courses of the true inputs $w_{3}^{*}\left(t\right)$ and $w_{6}^{*}\left(t\right)$ together with the input reconstruction by the recovery algorithm based on the minimization of the error functional (3), with β = 10^−2^. The strong coherence within the different clusters makes a reconstruction of the input impossible. **(C)** The input strength (7) for the different input clusters using the reconstructed input signals in **(A)**. Clearly, the input clusters $C_{1}$ and $C_{3}$ are reconstructed. These are also the clusters containing the true inputs at nodes $6\in C_{1}$ and $3\in C_{3}$.

We used a hierarchical clustering on the distance matrix for the input ground set L={1,…,11}. See Figure 2A for the influence graph of the system. The node coloring encodes the assignment to the six clusters

$$\begin{array}{l} C_{1}=\left\{6^{*},7,8\right\},C_{2}=\left\{4,10\right\},C_{3}=\left\{1,3^{*},5\right\},C_{4}=\left\{2\right\},C_{5}=\left\{9\right\},\\ C_{6}=\left\{11\right\}. \end{array}$$

The asterisk marks the input nodes that are affected by the model error.

To mimick a situation, where the location of the errors is unknown and has to be inferred from the output time courses *y*_1_(*t*), …, *y*_5_(*t*), we minimized the error functional (3) and obtained estimates for the unknown inputs *w*_1_, …, *w*_11_. We used the python implementation of the CasADi package for optimization and optimal control (Andersson et al., 2019). The regularization parameter can be chosen by the discrepancy method (Honerkamp and Weese, 1990; Engelhardt et al., 2016). One can also incorporate an invariable sparsity assumption and check, how many non-zero input nodes are needed to recover the output data $y_{1}^{\text{data}}\left(t\right),\ldots ,y_{5}^{\text{data}}\left(t\right)$ with sufficient accuracy.

As shown in Figure 2B, this unknown input reconstruction seems to be a failure. This is not caused by numerical problems, but by the high coherence of the states within the inputs clusters $C_{1},\ldots ,C_{6}$. States within one and the same cluster can in principle generate the same output functions, what prevents us from reconstructing inputs and their localization.

Nevertheless, let us compute an input strength for each cluster by summing the signal norms within each cluster according to

(7) $$\begin{array}{l} \|w_{C_{i}}{\|}_{1}:=\sum_{k\in C_{i}}{\left(\int_{0}^{T}|w_{k}(t){|}^{p}\text{d}t\right)}^{1/p}. \end{array}$$

In this paper, we chose *p* = 3, however, this parameter is theoretically arbitrary. The input strengths of each input cluster are plotted in Figure 2C with colors corresponding to the clusters shown in (A). Clearly, we can identify clusters $C_{1}$ and $C_{3}$ as the ones of highest input strength. These clusters indeed contain the true inputs nodes 3 and 6.

So, one strategy to narrow down the location of errors is to group the state nodes in the influence graph into input clusters based on their coherence. Then, we minimize (3) to compute the input strength (7) of these clusters. Now, we can see from the input strength, which clusters are most likely to be targeted by errors or other perturbations. We will further illustrate and extend this strategy in the next two examples.

### 3.3. Iterative Error Localization of an 1-Sparse Model Error

**Figure 3:**
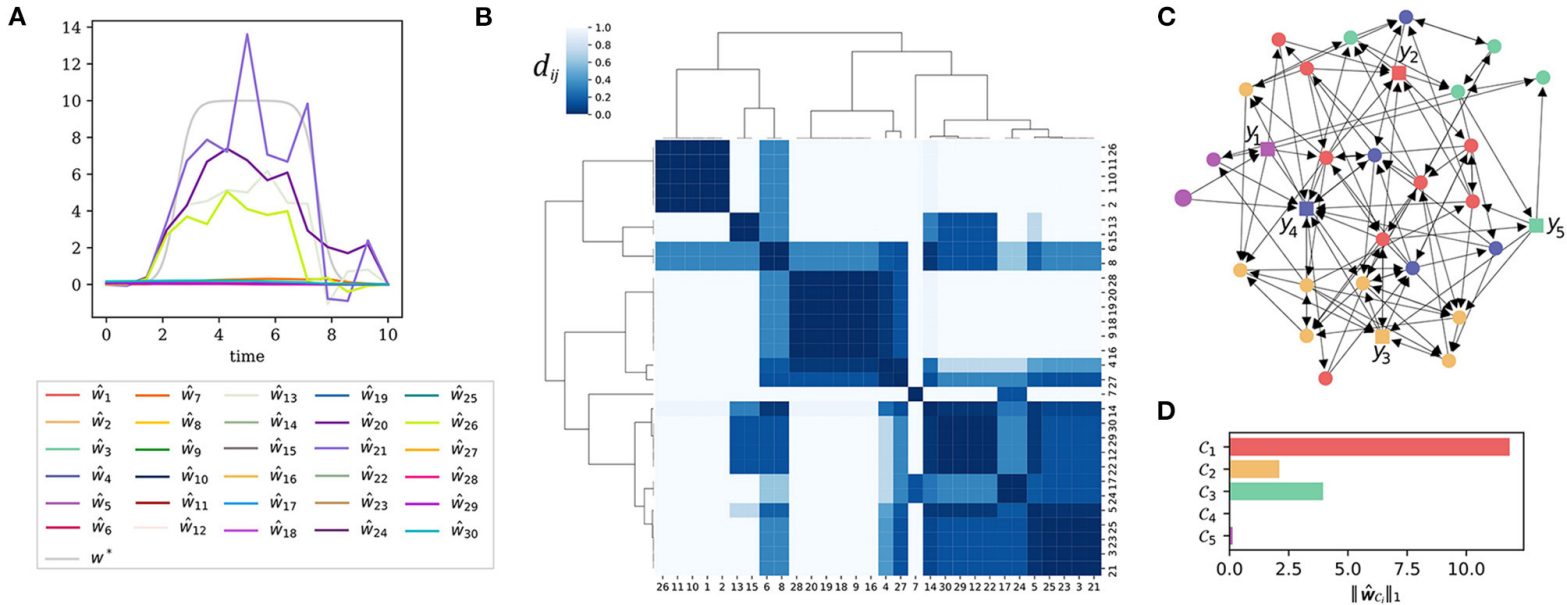
Error localization at the level of input clusters. **(A)** A direct application of the recovery algorithm Δ yields estimates ŵ_*i*_ for the model error, w^*^ shows the ground truth, β = 10^−2^. **(B)** The normalized distance matrix of the system presented as a heatmap. Highly coherent state nodes were grouped together by hierarchical clustering. **(C)** The influence graph of the system. The square shaped nodes represent the five outputs *y*_1_, …, *y*_5_. The node coloring encodes the affiliation to input clusters. We chose a clustering depth that produces five input clusters. **(D)** The total input strength (7) for each of the five input clusters.

In this and the next example, we consider a linear dynamic system of *N* = 30 state variables. The equations can be found in the Supplementary Material, the influence graph is also shown in Figure 3C. The system has five outputs *y*_1_, …, *y*_5_ as indicated by the square shaped output nodes in the figure. To simulate a structural model error, we added a single additional input to one of the differential equations of the system.

Our aim is to localize and recover this artificial model error. We will first apply the recovery algorithm and see that it does not lead to the desired outcome due to the high coherence of the input nodes. Next, we will cluster the input nodes by their coherence and identify the cluster targeted by the model error. To further narrow down the location of the error, we need a different set of output nodes. We show, that output nodes can be clustered in a similar way as inputs into groups of redundant outputs. Then, we can relocalize the sensor nodes to have non-redundant output measurements. The output clusters also allow to decide, whether more observables are needed. This enables us to iteratively narrow down the possible sources of the model error. Finally, we are able to pin down the location of the model error exactly and to compute an appropriate estimate of the ground truth error signal.

#### 3.3.1. Direct Recovery Attempt

Without prior knowledge about the model error, we must consider each of the *N* = 30 nodes to be a potential input node, hence the input ground set is $L_{0}:=\left\{1,\ldots ,30\right\}$. Again, we minimize the error function (3) to recover the unknown inputs. We will use a symbolic notation for this recovery algorithm, the expression

(8) $$\begin{array}{l} \Delta :\left(y_{1}^{\text{data}},\ldots ,y_{5}^{\text{data}}\right)\mapsto \left({ŵ}_{1}\ldots ,{ŵ}_{30}\right). \end{array}$$

means, that we use time course measurements $y_{1}^{\text{data}},\ldots ,y_{5}^{\text{data}}$ to reconstruct the outputs in the ground set $L_{0}:=\left\{1,\ldots ,30\right\}$.

As can be seen from Figure 3A, the result of this direct reconstruction attempt is not satisfactory.

#### 3.3.2. Localization of the Erroneous Cluster

Figure 3B reveals why the direct recovery attempt of the model error was bound to fail: In the (normalized) distance matrix (6) of the system one can see a strong cluster hierarchy. The output signal $y_{1}^{\text{data}}\left(t\right),\ldots ,y_{5}^{\text{data}}\left(t\right)$ can be caused by inputs from different nodes within one and the same input cluster. This makes a reconstruction of the model error impossible. Heuristically, we have found that a clustering into *P* clusters is usually a robust choice, where *P* is the number of sensors. In this example we work with *P* = 5 sensors.

In Figure 3C, we depict the influence graph of the system with the nodes colored according to the five input clusters of the system. The nodes within one and the same cluster are indistinguishable by the outputs of the system. Thus, it is impossible to localize the model error at a finer resolution than this given by the clusters. When computing the total input cluster strength (7), we see that the input to cluster $C_{1}$ is significantly larger than the input strength estimated for the other clusters (Figure 3D). Though we are not able to detect the model error exactly, we deduce that it must lie somewhere in cluster $C_{1}$.

#### 3.3.3. Sensor Replacement

Remember that we have computed the distance matrix for the input nodes using Γ ∘ Γ′ (see section 2.2.1). Due to the notion of the transposed gammoid we can also do a reverse action and compute a distance matrix for the output nodes via Γ′ ∘ Γ. More precisely, we perform the following procedure: Let M be an *output ground set*, i.e., the set of all nodes that can potentially be monitored. For this example, say M=N. Since we have already deduced a new input ground set $L_{1}$, we will work with $\tilde{\Gamma }=\left(C_{1},g,M\right)$ to compute a distance matrix for M.

**Figure 4:**
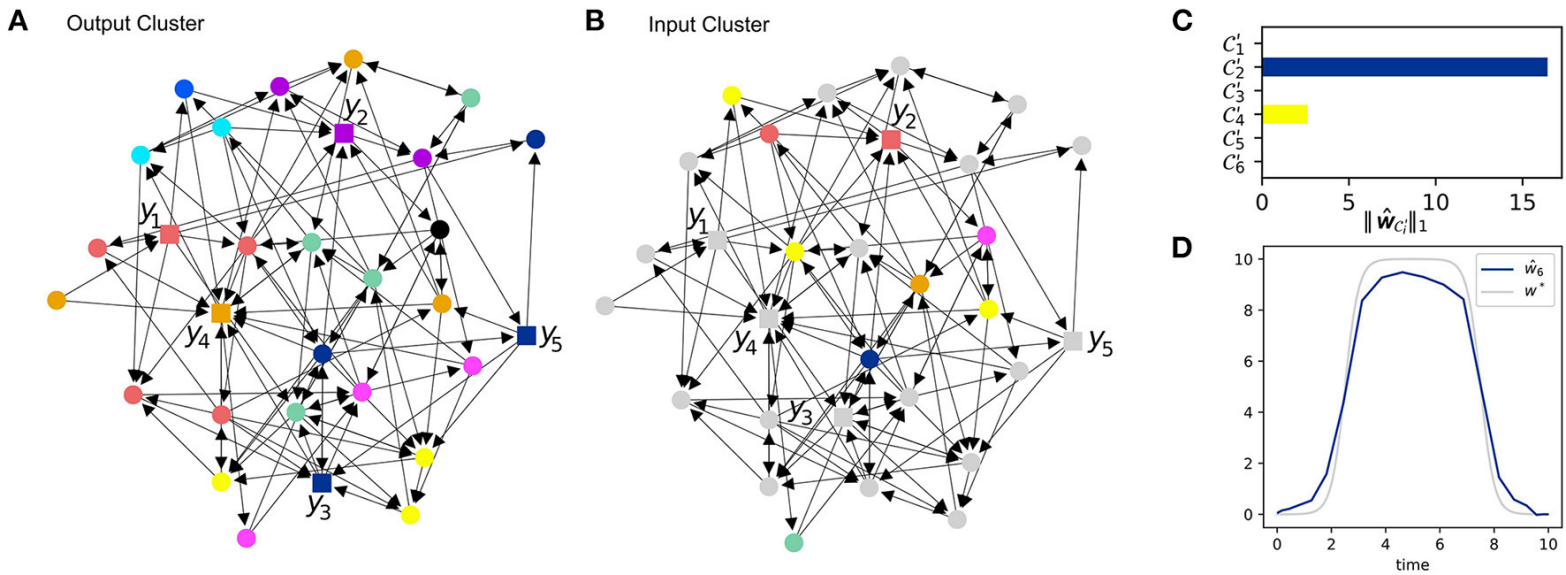
Iterative error reconstruction. **(A)** The influence graph of the same system as in Figure 3C. Here, the node coloring indicates *output* clusters. Measuring nodes within the same cluster provides only redundant information about errors. **(B)** The influence graph with node coloring indicating input clusters w.r.t the new ground set $C_{1}$ identified from the previous clustering in Figures 3C,D. The gray nodes do not lie in the input ground set and are therefore not considered. **(C)** The total input strength (7) for each new cluster in **(B)**. **(D)** The estimated model error ŵ_6_ compared to the ground truth w^*^, β = 0.1.

Output nodes with a high coherence provide redundant information, i.e., they can not help distinguishing inputs from different input nodes. See Figure 4A for the output clusters of the system. The output nodes *y*_3_ and *y*_5_ lie within the same output cluster. To enhance the informative value of our data, we replace *y*_3_ as indicated in Figure 4B such that it now covers a different output cluster.

#### 3.3.4. Final Localization and Recovery of the Model Error

We have already found that the model error is located in input cluster $C_{1}$. With this information we can define a new input ground set $L_{1}:=C_{1}$ and in combination with the less redundant output nodes we can try to further narrow down the source of the model error.

To this end, we perform again an input clustering using $L_{1}$ as ground set with the result shown in Figure 4B. Then, we apply the recovery algorithm Δ using the new output data and the (smaller) ground set $L_{1}:=C_{1}$. The cumulated input strength (7) for each cluster is shown in Figure 4C. It turns out that input cluster $C_{2}^{\prime }$ plays the predominant role so that we again reduce the input ground set and obtain $L_{2}:=C_{2}^{\prime }$.

The new input ground set $L_{2}$ consists of only one node. Hence, we have pinned down the source of the model error to only one possible input node. Figure 4D presents the estimate obtained from Δ with $L_{2}$ as input ground set as well as the ground truth. The accuracy of the estimate clearly relies on the chosen recovery algorithm Δ as well-stochastic uncertainties in the data.

### 3.4. Iterative Error Localization of a 2-Sparse Model Error

As another example, let us again consider the same *N* = 30 model as before. Now, we add two artificial model errors affecting state variables *x*_6_ and *x*_30_. Again, these nodes are not chosen with any preference and the same procedure will work comparably for other choices. However, with more input nodes, the high coherence and indistinguishability will diminish the ability to localize and reconstruct the model errors. In this example of two model errors, we will see that the number of sensor nodes is too small to obtain an accurate estimate. Still, we will be able to narrow down the set of potential inputs to a much smaller set using the same number of sensors.

#### 3.4.1. Direct Recovery Attempt

**Figure 5:**
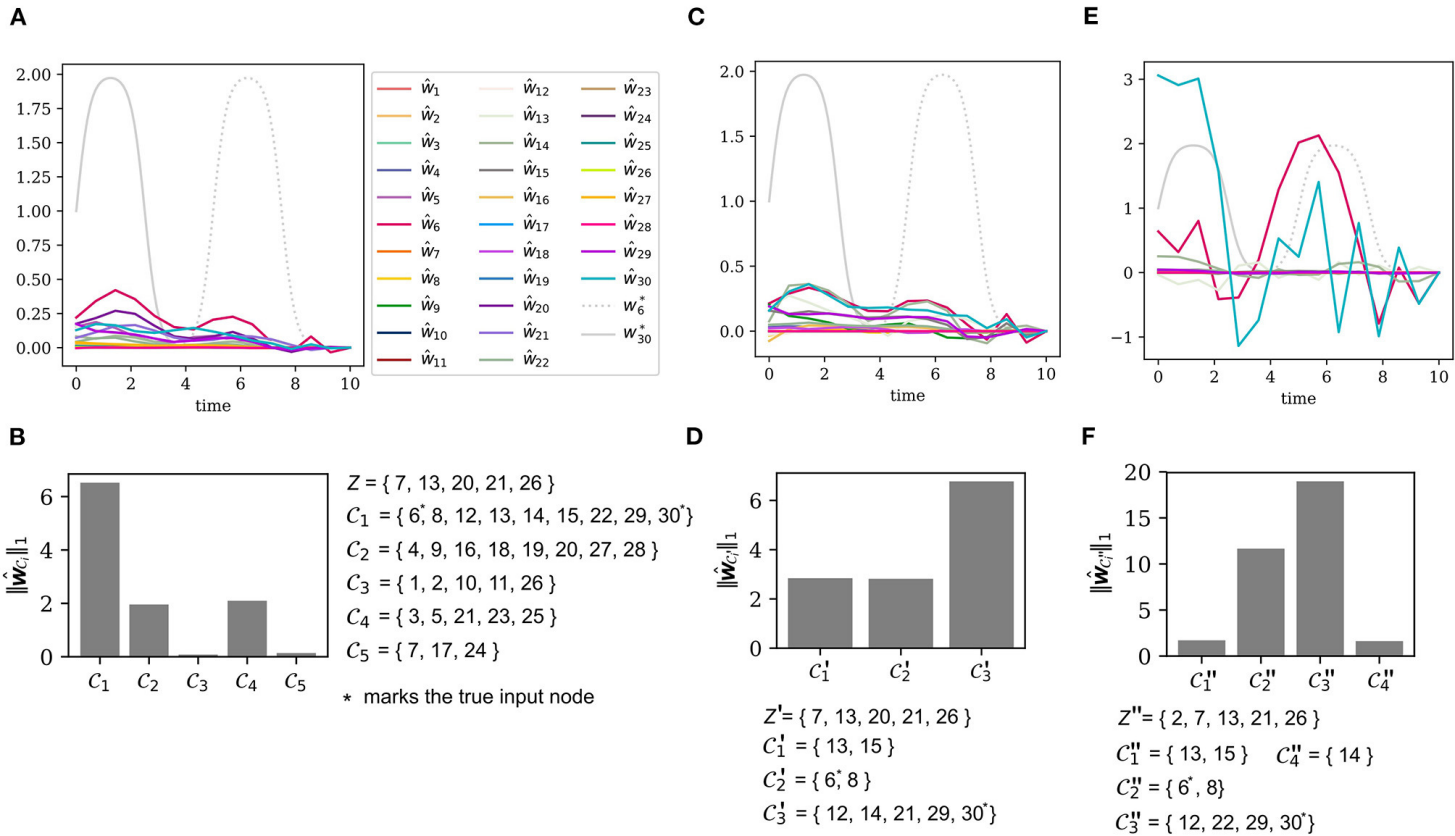
Iterative error reconstruction for the same system as in Figure 3, but now with two unknown inputs. **(A)** The true model errors $w_{6}^{*}$ and $w_{30}^{*}$ and the input estimates ŵ_1_, …, ŵ_30_ for the sensor as described by the set *Z* (see **B**), β = 0.1. Each node was considered a potential input node. (B) The total input strength for each of the input clusters $C_{1},\ldots ,C_{5}$ w.r.t. the output set *Z*. **(C)** The true and estimated inputs, where only nodes from $L_{1}=C_{1}$ were considered as potential input nodes, again β = 0.1. The output set remained the same, i.e., *Z*′ = *Z*. **(D)** The total input strength for each of the new clusters $C_{1}^{\prime },C_{2}^{\prime },C_{3}^{\prime }$, when the ground set is restricted to $L_{1}=C_{1}$, but the sensor locations *Z*′ = *Z* remain the same. **(E)** The true and estimated inputs, where only nodes from $L_{1}=C_{1}$ were considered as potential input nodes, but the new output set *Z*″ was chosen to cover distinct clusters, again β = 0.1. **(F)** The total input strength derived from **(E)** for the new input clusters $C_{1}^{\prime \prime },\ldots ,C_{6}^{\prime \prime }$ w.r.t. the less coherent output set *Z*″.

Without prior knowledge, each of the *N* = 30 nodes is considered a potential input node, hence the input ground set is again $L_{0}:=\left\{1,\ldots ,30\right\}$. The sensors are placed at the output node set *Z* = {7, 13, 20, 21, 26}. Figure 5A shows the ground truth as well as the result of the direct error reconstruction. Due to thigh coherence of input nodes (compare Figures 3B,C) the input estimates do not approximate the ground truth.

#### 3.4.2. Detection of the Erroneous Clusters

Figure 5B shows the input cluster strength (7) for each of the five input clusters $C_{1},\ldots ,C_{5}$. Cluster $C_{1}$ plays the dominant role so that we chose the new input ground set to be $L_{1}:=C_{1}$.

#### 3.4.3. The Need for Sensor Replacement

Let us first see what happens, if we are not able to replace the sensors. Figure 5C shows the input estimates with the unchanged output set *Z*′ = *Z* = {7, 13, 20, 21, 26}. A clustering of the adjusted input ground set leads to three clusters $C_{1}^{\prime }$, $C_{2}^{\prime }$, and $C_{3}^{\prime }$. Figure 5D shows the total input strength for each cluster. Clearly, the input estimates do not describe the ground truth appropriately and the input strength of each cluster is also not informative about the true location of the error.

As the new input ground set $L_{1}$ has already been identified as the target of the perturbations, it is not surprising that the input estimation for the large input ground set $L_{0}$ (see Figure 5A) and for the reduced ground set $L_{1}$ (see Figure 5C) produce nearly the same result. Since the sensor set is the same as before, *Z*′ = *Z*, the measurements suffer from the high coherence of the input nodes and Figure 5D shows that we are unable to improve the localization of the inputs.

#### 3.4.4. Localization With Sensor Replacement

As seen in the previous example, the sensor set *Z* is not a good choice, because the observables monitoring *x*_20_ and *x*_26_ yield redundant information. We exchange the sensor on *x*_20_ with a sensor measuring *x*_2_. Again, choosing the new sensor is subject to practical issues, e.g., in an experiment. From the theoretical point of view, we should just try to place the sensors such that they cover different input clusters. We change the output set to *Z*″ = {2, 7, 13, 21, 26}. The new input estimates can be found in Figure 5E. One will see that the estimates shown there come closer to the ground truth but are not accurate. The new output set *Z*″ implies a new clustering of the input ground set $L_{1}$ into $C_{1}^{\prime \prime },\ldots ,C_{4}^{\prime \prime }$ as presented in Figure 5F. The input strength for each of the clusters indicates that the two clusters $C_{2}^{\prime \prime }$ and $C_{3}^{\prime \prime }$ have the largest total input strength. Indeed, these two clusters contain the nodes 6 and 30, which are targeted by the added perturbations.

Though we were unable to accurately reconstruct the true model errors, we have still succeeded to narrow down the list of potentially perturbed states. With an initial ground set $L_{0}$ of 30 nodes and only five sensors, the system is highly under-determined. With an iterative input estimation and sensor replacement we have found that the perturbations lie within the much smaller clusters $C_{2}^{\prime \prime }$ of size two and $C_{3}^{\prime \prime }$ of size four. Thus, we can exclude the remaining 24 nodes and declare them as non-perturbed. A further reduction would only be possible with a higher number of output sensors.

## 4. Discussion

Developing sufficiently accurate models for large and complex dynamic networks is often difficult because we neither know all the details about the endogenous interactions in the system nor can we be sure that the system acts in isolation. This limited information inevitably causes structural model errors which include misspecified couplings, system faults as well as unknown inputs from the exterior. Localizing and identifying these errors is a crucial step toward better estimates for the current and future behavior of a system and to reliable mathematical models.

We have presented a coherence measure for dynamic networks, which indicates how difficult it is to decide for two different nodes, whether an error targets the one or the other node. This coherence is based on a weighted gammoid representation of the dynamic system and can efficiently be computed even for very large dynamic systems. The coherence can be used to cluster network nodes into groups of state variables which can not be distinguished as potential sources of error. By combining this clustering with an optimization based inference of cluster inputs, we are able to localize structural model errors and unknown inputs down to the level of these input clusters. We demonstrated for the *C. elegans* neural network that this approach can identify meaningful input clusters which we predict to correspond to the four different movements previously reported (Stephens et al., 2008). We would like to emphasize, that our coherence measure can be used for any directed weighted network with known output nodes.

By using the dual approach for sensor nodes, we can identify non-redundant sensors which can be used to further narrow down the exact position of the error. This motivates an algorithm iterating between input clustering, output clustering and sensor node selection. We demonstrated that this procedure can efficiently select non-redundant measurements. If there are enough sensors, it can even be possible to finally pinpoint the exact location of an unknown input. In other cases, when the number of outputs is not sufficient, it might be only possible to reduce the possible nodes to a smaller set. Please note, however, that one limitation of our work is the lack of an exact proof for the convergence of this iterative procedure. This is left as a direction for future research.

The localization and reconstruction of errors and unknown inputs in a model of a dynamic system is a crucial step to systematically extend models. If we know, where a model is incorrect, we can systematically improve it. An interesting question is, how to best combine model error analysis with data driven model discovery (Brunton et al., 2016). Despite the recent progress (Brunton et al., 2016) in model discovery, it is likely that the data sets required for a *de novo* reconstruction of the governing equations of a model will not always be available in biology, medicine, or physiology. Thus, we believe that a combination of modeling, data driven model error reconstruction, and data driven model extension will be the most promising approach toward an understanding of complex dynamic systems in the biomedical field.

## Data Availability Statement

The original contributions presented in the study are included in the article/Supplementary Material, further inquiries can be directed to the corresponding author/s.

## Author Contributions

DK performed the research and computational analysis. MK designed and performed the research. DK and MK wrote the paper. Both authors contributed to the article and approved the submitted version.

## Conflict of Interest

The authors declare that the research was conducted in the absence of any commercial or financial relationships that could be construed as a potential conflict of interest.
