# Supplementary material for: Searching for Errors in Models of Complex Dynamic Systems
Source: Front Physiol. 2021 Jan 11;11:612590. doi: 10.3389/fphys.2020.612590 (PMC7830364; doi:10.3389/fphys.2020.612590)
Supplement: Supplementary file 1 [file Data_Sheet_1.PDF]

# Supplementary Material

## 1 EQUATIONS OF THE UV-B SYSTEM

The equations of the UV-B model can be found in Ouyang et al. (2014) and are given below.

$$\begin{aligned}
\dot{x}_1 =& -2ka_1x_1^2x_4^2 + 2kd_1x_5 + ks_1\,(1 + UV\ n_3\ x_9 + FHY3) \\
& - kdr_1\,(1 + (n_1UV))\,x_1 - kd_2x_3 - 2ka_2x_1^2x_2 \\
\dot{x}_2 =& -ka_2x_1^2x_2 + kd_2x_3 + ka_4x_2x_{10} + kd_4x_{11} \\
\dot{x}_3 =& -kd_2x_3 + ka_2x_1^2x_2 \\
\dot{x}_4 =& -2k_1x_4^2 + 2k_2x_6 - 2ka_1x_1^2x_4^2 + 2kd_1x_5 - ka_3x_4x_7 \\
\dot{x}_5 =& -kd_1x_5 + ka_1x_1^2x_4^2 \\
\dot{x}_6 =& -k_2x_6 + k_1x_4^2 + kd_3x_8^2 \\
\dot{x}_7 =& -ka_3x_4x_7 + ks_2\,(1 + UVx_5) - kdr_2x_7 + 2kd_3x_8^2 \\
\dot{x}_8 =& -2kd_3x_8^2 + ka_3x_4x_7 \\
\dot{x}_9 =& -kdr_3\left(\frac{x_3}{kdr_{3a} + x_3} + \frac{x_{11}}{kdr_{3b} + x_{11}}\right)x_9 \\
& + ks3_p\,(1 + n_2UV) - kdr3\left(\frac{x_5}{ksr + x_5}\right)x_9 \\
\dot{x}_{10} =& -ka_4x_2x_{10} + kd_4x_{11} \\
\dot{x}_{11} =& -kd_4x_{11} + ka_4x_2x_{10}\,.
\end{aligned}$$

The observable quantities are

$$y_1 = 2x_5 + x_4 + x_8; \qquad y_3 = x_6; \qquad y_2 = 2x_5 + 2x_3 + x_1$$
$$y_4 = x_9; \qquad y_5 = x_4\,.$$

The parameters are given by

$$\begin{aligned}
ks_1 &= 0.23; & ks_2 &= 4.0526; & k_1 &= 0.0043; \\
kdr_1 &= 0.1; & kdr_2 &= 0.2118; & k_2 &= 161.62; \\
ka_1 &= 0.0372; & ka_2 &= 0.0611; & ka_3 &= 4.7207; \\
kd_2 &= 50.6973; & kd_3 &= 0.5508; & kd_1 &= 94.3524; \\
ks_3 &= 0.4397; & kdr_3 &= 1.246; & UV &= 1; \\
kd_4 &= 1.1999; & n_1 &= 3; & ka_4 &= 10.1285; \\
n_2 &= 2; & n_3 &= 3.5; & kdr_{3a} &= 0.9735; \\
ksr &= 0.7537; & FHY3 &= 5; & kdr_{3b} &= 0.406\,.
\end{aligned}$$

The initial value given in Ouyang et al. (2014) is

$$\tilde{\boldsymbol{x}}_0 = (0.2, 10.0, 2.0, 0.0, 0.0, 20.0, 0.0, 0.0, 0.25, 20.0, 0.0)^T .$$

At around $\tilde{t} = 0.25$ the system runs slowly into equilibrium. In this example, we are interested in perturbations from this state at $\tilde{t} = 0.25$, so we define the state at $\tilde{t} = 0.25$ as initial value

$$\boldsymbol{x}_0 = (1.89, 0.17, 0.0007, 34.34, 1.63, 0.048, 0.098, 2.27, 0.40, 8.17, 11.82)^T \tag{S1}$$

and set the initial time $t = 0$.

## 2 EQUATIONS OF THE LINEAR SYSTEM

In the examples 3.3 and 3.4 of the main text we follow an iterative error localization strategy to narrow down the location of a model error. The nominal model comprises $N = 30$ state variables according to the differential equations

$$\dot{x}_1(t) = x_7(t) + x_{26}(t) - \alpha x_1(t)$$
$$\dot{x}_2(t) = x_3(t) + x_{14}(t) - \alpha x_2(t)$$
$$\dot{x}_3(t) = x_2(t) + x_9(t) + x_{11}(t) + x_{22}(t) + x_{30}(t) - \alpha x_3(t)$$
$$\dot{x}_4(t) = x_6(t) + x_{14}(t) + x_{16}(t) + x_{21}(t) + x_{28}(t) - \alpha x_4(t)$$
$$\dot{x}_5(t) = x_8(t) + x_{16}(t) + x_{19}(t) + x_{20}(t) + x_{29}(t) - \alpha x_5(t)$$
$$\dot{x}_6(t) = x_8(t) + x_{14}(t) + x_{15}(t) + x_{20}(t) + x_{30}(t) - \alpha x_6(t)$$
$$\dot{x}_7(t) = x_{17}(t) + x_{24}(t) - \alpha x_7(t)$$
$$\dot{x}_8(t) = x_{21}(t) - \alpha x_8(t)$$
$$\dot{x}_9(t) = x_{15}(t) + x_{21}(t) + x_{30}(t) - \alpha x_9(t)$$
$$\dot{x}_{10}(t) = x_1(t) + x_2(t) + x_{11}(t) + x_{13}(t) - \alpha x_{10}(t)$$
$$\dot{x}_{11}(t) = x_9(t) + x_{13}(t) + x_{14}(t) + x_{21}(t) - \alpha x_{11}(t)$$
$$\dot{x}_{12}(t) = x_7(t) + x_{14}(t) - \alpha x_{12}(t)$$
$$\dot{x}_{13}(t) = x_6(t) + x_{12}(t) + x_{15}(t) + x_{22}(t) + x_{30}(t) - \alpha x_{13}(t)$$
$$\dot{x}_{14}(t) = x_2(t) + x_{16}(t) + x_{18}(t) + x_{29}(t) - \alpha x_{14}(t)$$
$$\dot{x}_{15}(t) = x_{23}(t) - \alpha x_{15}(t)$$
$$\dot{x}_{16}(t) = x_5(t) + x_7(t) + x_{14}(t) + x_{20}(t) + x_{23}(t) + x_{28}(t) - \alpha x_{16}(t)$$
$$\dot{x}_{17}(t) = -\alpha x_{17}(t)$$
$$\dot{x}_{18}(t) = x_{16}(t) + x_{25}(t) + x_{27}(t) + x_{28}(t) - \alpha x_{18}(t)$$
$$\dot{x}_{19}(t) = x_4(t) + x_7(t) + x_9(t) + x_{27}(t) + x_{30}(t) - \alpha x_{19}(t)$$
$$\dot{x}_{20}(t) = x_6(t) + x_{18}(t) + x_{19}(t) + x_{26}(t) + x_{27}(t) - \alpha x_{20}(t)$$
$$\dot{x}_{21}(t) = x_6(t) + x_{12}(t) + x_{17}(t) + x_{20}(t) + x_{22}(t) + x_{23}(t) + x_{24}(t) + x_{25}(t) + x_{27}(t) + x_{30}(t) - \alpha x_{21}(t)$$
$$\dot{x}_{22}(t) = x_{26}(t) + x_{27}(t) + x_{29}(t) - \alpha x_{22}(t)$$
$$\dot{x}_{23}(t) = x_3(t) + x_5(t) + x_{14}(t) + x_{21}(t) + x_{25}(t) + x_{30}(t) - \alpha x_{23}(t)$$
$$\dot{x}_{24}(t) = x_{10}(t) + x_{27}(t) - \alpha x_{24}(t)$$
$$\dot{x}_{25}(t) = x_5(t) + x_{10}(t) - \alpha x_{25}(t)$$
$$\dot{x}_{26}(t) = x_6(t) + x_{10}(t) - \alpha x_{26}(t)$$
$$\dot{x}_{27}(t) = x_4(t) + x_{30}(t) - \alpha x_{27}(t)$$
$$\dot{x}_{28}(t) = x_6(t) + x_{14}(t) + x_{16}(t) + x_{22}(t) + x_{26}(t) + x_{27}(t) - \alpha x_{28}(t)$$
$$\dot{x}_{29}(t) = x_5(t) + x_{13}(t) + x_{22}(t) - \alpha x_{29}(t)$$
$$\dot{x}_{30}(t) = x_5(t) + x_7(t) + x_{15}(t) + x_{29}(t) - \alpha x_{30}(t)$$

Here, $\alpha = 4$ is a good choice to render the system stable so that we can focus on the coherence structure of the system without getting distracted by numerical issues. The initial value is chosen as $\boldsymbol{x}_0 = 0 \in \mathbb{R}^{30}$.

### 2.1 1-Sparse Model Error

Let us first focus on the situation from example 3 with one model error $w_6^*$ in the equation for $x_6$,

$$\tilde{\boldsymbol{x}}_6 = \tilde{x}_8(t) + \tilde{x}_{14}(t) + \tilde{x}_{15}(t) + \tilde{x}_{20}(t) + \tilde{x}_{30}(t) - \alpha \tilde{x}_6(t) + w_6^*(t) . \tag{S2}$$

Here, the tilde-sign indicates the *real system* which we use to generate pseudo-experimental data. As perturbation we used

$$w_6^*(t) = 5(\tanh(-2t + 15) - \tanh(-2t + 5)) . \tag{S3}$$

This function produces a smooth and differentiable pulse as a place holder for some unknown external input. Finally, we added Gaussian noise with relative standard deviation of $\sigma = 5\%$ for each data point in order to simulate measurement noise.

The initial observables of the system are given by $\boldsymbol{y}(t) = \boldsymbol{c}(\boldsymbol{x}(t))$ where

$$\boldsymbol{c}(\boldsymbol{x}) = (x_7, x_{13}, x_{20}, x_{21}, x_{26})^T \tag{S4}$$

which can be represented by the output set

$$Z_0 = \{7, 13, 20, 21, 26\}\,. \tag{S5}$$

We find the output clusters shown in figure 4(A). Since $y_3$ and $y_5$ lie in the same cluster, we define the new output set as

$$Z_1 = \{7, 13, 16, 21, 26\}\,, \tag{S6}$$

i.e., we replace $y_3 = x_{20}$ with $y_3 = x_{16}$.

## 2.2 2-Sparse Model Error

Example 3.4 builds on the same nominal model of $N = 30$ coupled differential equations. We perturb the system with

$$w_6^* = \tanh(-2t + 15) - \tanh(-2t + 10) \tag{S7}$$

and

$$w_{30}^* = \tanh(-2t + 5) - \tanh(-2t)\,. \tag{S8}$$

We start again with the output set

$$Z = \{7, 13, 20, 21, 26\} \tag{S9}$$

and figure 5(B) shows that again input cluster $\mathcal{C}_1$ is dominant. Note that $Z = Z_0$ from the 1-sparse example, thus the input clusters are the same as presented in figure 3(B,C).

To see how the sensor replacement, i.e., choosing the output set so that distinct output clusters are covered, we perform the next iteration step in two versions.

First, we do not care about the coherence of $y_3$ and $y_5$ shown in figure 4(B) and keep the output set

$$Z' = Z\,. \tag{S10}$$

Second, we define a new output set as

$$Z'' = \{2, 7, 13, 21, 26\} \tag{S11}$$

i.e., we replace $y_3 = x_{20}$ with $y_3 = x_2$.

# 3 A NOTE ON THE COST FUNCTIONAL

In the cost functional

$$J[\boldsymbol{w}] = \frac{1}{2}\|\boldsymbol{y}^{\text{data}} - \Phi(\boldsymbol{w})\|_2^2 + \beta\|\boldsymbol{w}\|_1 \tag{S12}$$

we use a combined $p$-$q$-norm defined by

$$\|\boldsymbol{w}\|_q := \left( \left[ \int_0^T |w_i|^p \, \mathrm{d}t \right]^{q/p} \right)^{1/q} \tag{S13}$$

as presented in (Kahl et al., 2020). The parameter $p$ is suppressed in the notation for it was shown that it can be chosen arbitrarily between $1 \leq p < \infty$. We choose $p = 3$ throughout the paper.

## REFERENCES

Kahl, D., A. Weber, and M. Kschischo (2020). Sparse error localization in complex dynamic networks.

Ouyang, X., X. Huang, X. Jin, Z. Chen, P. Yang, H. Ge, S. Li, and X. W. Deng (2014, August). Coordinated photomorphogenic UV-B signaling network captured by mathematical modeling. *Proc. Natl. Acad. Sci. U.S.A. 111*(31), 11539–11544. 00003.
